# Effect of cigarette smoke on the proliferation, viability, gene expression, and cellular functions of adipose-derived mesenchymal stem cells from smoking and non-smoking donors

**DOI:** 10.1242/bio.061665

**Published:** 2024-12-03

**Authors:** Bareqa Salah, Diana Shahin, Momen Sarhan, Joud Al-Karmi, Ban Al-Kurdi, Renata Al-Atoom, Mohammad A. Ismail, Nouran Hammad, Hanan Jafar, Abdalla Awidi, Nidaa A. Ababneh

**Affiliations:** ^1^ General Surgery Department/Plastic & Reconstructive, Jordan University Hospital, the University of Jordan, 11942; ^2^ Cell Therapy Center, the University of Jordan, Amman, Jordan, 11942; ^3^ School of Medicine, the University of Jordan, Amman, Jordan, 11942; ^4^ School of Medicine, Jordan University of Science and Technology, Al-Ramtha, Jordan, 22110; ^5^ Hemostasis and Thrombosis Laboratory, School of Medicine, the University of Jordan, Amman, Jordan, 11942; ^6^ Department of Hematology and Oncology, Jordan University Hospital, Amman, Jordan, 11492

**Keywords:** Cigarette smoking, Adipose-derived MSCs (ADMSCs), Oxidative stress, Stem cells

## Abstract

Cigarette smoking negatively impacts mesenchymal stem cell functionality, including proliferation, viability, and differentiation potential. Adipose-derived mesenchymal stem cells (ADMSCs) are increasingly used for therapeutic purposes, but the specific effects of smoking *in vivo* on these cells are poorly understood. This study investigates the effects of cigarette smoke on the proliferation, viability, gene expression, and cellular functions of ADMSCs from smoking and non-smoking donors. In this study, ADMSCs were isolated from healthy smokers and non-smokers, and cell proliferation was assessed using the MTT assay, viability with apoptosis assays, mitochondrial membrane potential (MMP), and gene expression related to oxidative stress and cellular functions. Cell cycle analysis was also conducted. Our findings reveal a significant decrease in the proliferation of ADMSCs from smokers. Apoptosis assays showed reduced viable cells in smokers without a significant change in MMP, suggesting alternative pathways contributing to decreased viability. Gene expression analysis indicated the upregulation of genes associated with oxidative stress response and cellular defense mechanisms and the downregulation of genes related to inflammatory signaling, detoxification, and cellular metabolism. Cell cycle analysis indicates cycle arrest or delay in smokers, possibly due to stress and potential DNA damage. Smoking negatively affects ADMSCs’ proliferation, viability, and function through oxidative stress and gene expression alterations. These findings highlight the importance of considering smoking status in ADMSC therapies and the need for further research to mitigate the effect of smoking on stem cells.

## Teaser

**Summary:** Smoking impairs adipose-derived mesenchymal stem cell (ADMSC) functionality, reducing proliferation and viability, altering oxidative stress responses, and diminishing therapeutic potential.

## INTRODUCTION

Mesenchymal stem cells (MSCs) are multipotent stem cells that can differentiate into multiple cell lineages, including adipocytes, chondrocytes, and osteocytes (Pittenger et al., 1999). Bone marrow remains the most valuable source of MSCs. However, adipose tissue has been increasingly used due to its high abundance of MSCs and the less invasive isolation procedures (Naji et al., 2019). MSCs can also be isolated from other tissues, including skin, dental pulp and perinatal tissues such as the umbilical cord, placenta, and amniotic fluid (Witkowska-Zimny and Wrobel, 2011; Giai Via et al., 2012).

MSCs exhibit several key functional properties, including the ability to home into native niches and to migrate into damaged tissues (Karp and Leng Teo, 2009), multipotency which is manifested by their ability to differentiate into various mesodermal cell lineages (Chamberlain et al., 2007), secretion of growth factors, chemokines, interleukins and extracellular matrix molecules (Majumdar et al., 2000; Hofer and Tuan, 2016), which aid in the maintenance, expansion and differentiation of hematopoietic stem cells (HSCs) within their niches *in vivo*, as well as other progenitor cells (Maitra et al., 2004; Caplan, 2007). MSCs also modulate innate and adaptive immunity by suppressing a broad range of immune cells (Gao et al., 2016).

The biological properties of MSCs allow them to be a valuable tool in a wide range of clinical applications. Alone or in combination with other drugs, MSCs have been used in the treatment of degenerative diseases affecting different organs, such as macular degeneration and retinitis pigmentosa of the eye (Limoli et al., 2016; Oner et al., 2016), acute kidney injury (Humphreys and Bonventre, 2008) and ischemic cardiomyopathy (Karantalis and Hare, 2015). Furthermore, the immunosuppressive properties of MSCs rendered them beneficial in treating and managing autoimmunity and immune rejection, including conditions such as Crohn's disease, rheumatoid arthritis, HSC transplantation, and solid organ transplantation (Gao et al., 2016). Ensuring the safety and efficacy of MSC-based therapies necessitates stringent adherence to good manufacturing practice (GMP) standards, which address factors like sterility and genetic stability (Sensebé et al., 2013). One of the risk factors that is often overlooked is the exposure of MSCs to cigarette smoke or nicotine, which may lead to less favorable clinical outcomes (Greenberg et al., 2017).

Cigarette smoking and nicotine use have the potential to impair the regenerative capacity of MSCs (Greenberg et al., 2017). Smoking has been shown to negatively affect MSC proliferation, possibly through one of two mechanisms: the production of reactive oxygen species (ROS) leading to oxidative stress in bone marrow MSCs (BM-MSCs) (Shen et al., 2013) and nicotine-induced cell cycle changes in human umbilical cord MSCs, as nicotine has been demonstrated to increase the G0/G1 ratio, thereby slowing cell division (Zeng et al., 2014). Additionally, the migratory potential of MSCs is significantly reduced following exposure to either cigarette smoke in periodontal ligament-derived stem cells (PDLSCs) (Ng et al., 2015) or nicotine in both adult human MSCs and PDLSCs (Ng et al., 2013) *in vitro*. Smoking also alters the paracrine secretion profile of MSCs, as shown in adipose-derived MSCs (ADMSCs), reducing the secretion of key cytokines such as IL-6 and IL-8, which in turn affects the immunomodulatory properties of MSCs (Wahl et al., 2016). Consequently, the collective impact of smoking severely restricts the potential of MSCs to aid in tissue repair and regeneration.

Although cigarette smoke has been found to alter MSC properties, limited research has examined its effect on adipose tissue-derived mesenchymal stem cells (ADMSCs). Importantly, most existing studies have relied on *in vitro* models or animal studies, where cells are artificially exposed to smoke, which may not fully replicate the complexities of *in vivo* exposure. Our research, however, focuses on human ADMSCs directly isolated from smokers, offering a more accurate representation of the chronic effects of smoking on these cells. This study aims to investigate the effect of cigarette smoke exposure on adipose tissue-derived MSCs and explore the potential differences in migratory capacity, differentiation potential, and proliferative ability of ADMSCs isolated from smokers and non-smokers.

## RESULTS

### Morphological characterization of ADMSCs

**Fig. 1.**
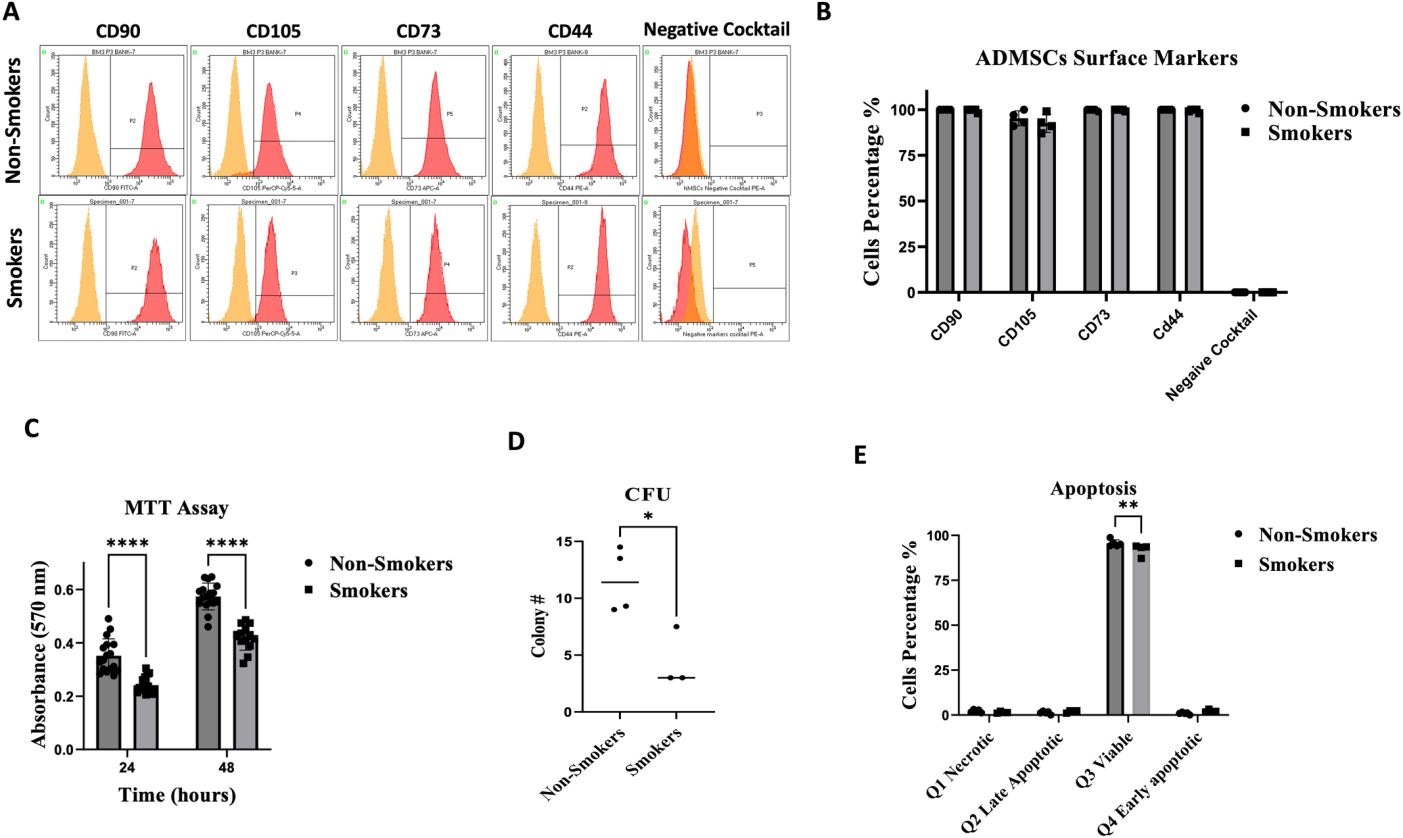
**Flow cytometric characterization, cell viability and metabolic activity measurement of ADMSCs by MTT, CFU and apoptosis assays.** (A,B) Flow cytometric analysis of ADMSCs from smoking and non-smoking positive (CD90, CD105, CD73, and CD44) and negative (CD34, CD11b, CD19, CD45 and HLA-DR) surface markers. (C) ADMSC metabolic activity was measured by MTT assay after 24 and 48 h of seeding. The results showed significant differences in the metabolic activity between the smoking and non-smoking groups after 24 (****P<0.0001) and 48 h (****P<0.0001). (D) The percentage of clonogenic cells among smoking and non-smoking ADMSCs. The results demonstrated significantly higher colony-forming units (CFU) among the non-smoking ADMSCs (*P<0.05). (E) The apoptosis assay percentage of viable cells was significantly higher in the non-smoking group 95.6% (±1.9) compared to the smoking group 92.0% (±3.2) (**P<0.01).

Adipose tissue-derived MSCs were isolated from both smoker and non-smoker participants. In both groups, these cells exhibited a fibroblast-like morphology and adhered to plastic surfaces when observed under an inverted phase-contrast microscope. No discernible morphological distinctions were observed between the two groups (Fig. S1). Flow cytometric analysis of surface marker proteins of both smoker and non-smoker MSCs showed positive expression (≥95% positive) of mesenchymal stem cells markers (CD90, CD73, CD44, and CD105) and negative expression (≤2% positive) of hematopoietic markers (CD34, CD11b, CD19, CD45, and HLA-DR), confirming the identity of MSCs with no significant difference between the two groups (Fig. 1A&B).

### Cell viability

To evaluate the viability and metabolic activity of adipose tissue-derived MSCs from smokers and non-smokers, an MTT assay was conducted at 24-, and 48 h post-cell seeding. Results revealed a notable difference in relative absorbance measurements between the two groups. Specifically, the smoking group exhibited significantly lower metabolic activity compared to the non-smoking group at both 24 h (*P*≤0.0001) and 48 h (*P*≤0.0001) post-seeding, indicating reduced metabolic activity in ADMSCs from smokers (Fig. 1C).

Smoking harmed the colony-forming abilities of ADMSCs. Colony forming units (CFU) assay showed that cells derived from smokers exhibited a significant decrease (*P*≤0.05) in their capacity to proliferate and form colonies compared to those derived from non-smokers (Fig. 1D).

### Apoptosis assessment

To evaluate the proportions of apoptotic, necrotic, and viable cells between the adipose-derived MSC groups of smokers and non-smokers, Annexin V protein was used to assess the viable cell percentages by flow cytometry. Findings revealed no significant difference in the percentages of apoptotic and necrotic cells. However, a significantly higher percentage of viable cells was observed in the non-smoking group compared to the smoking group (*P*≤0.001) (Fig. 1E)

### Cell cycle analysis, ROS detection and MMP

**Fig. 2.**
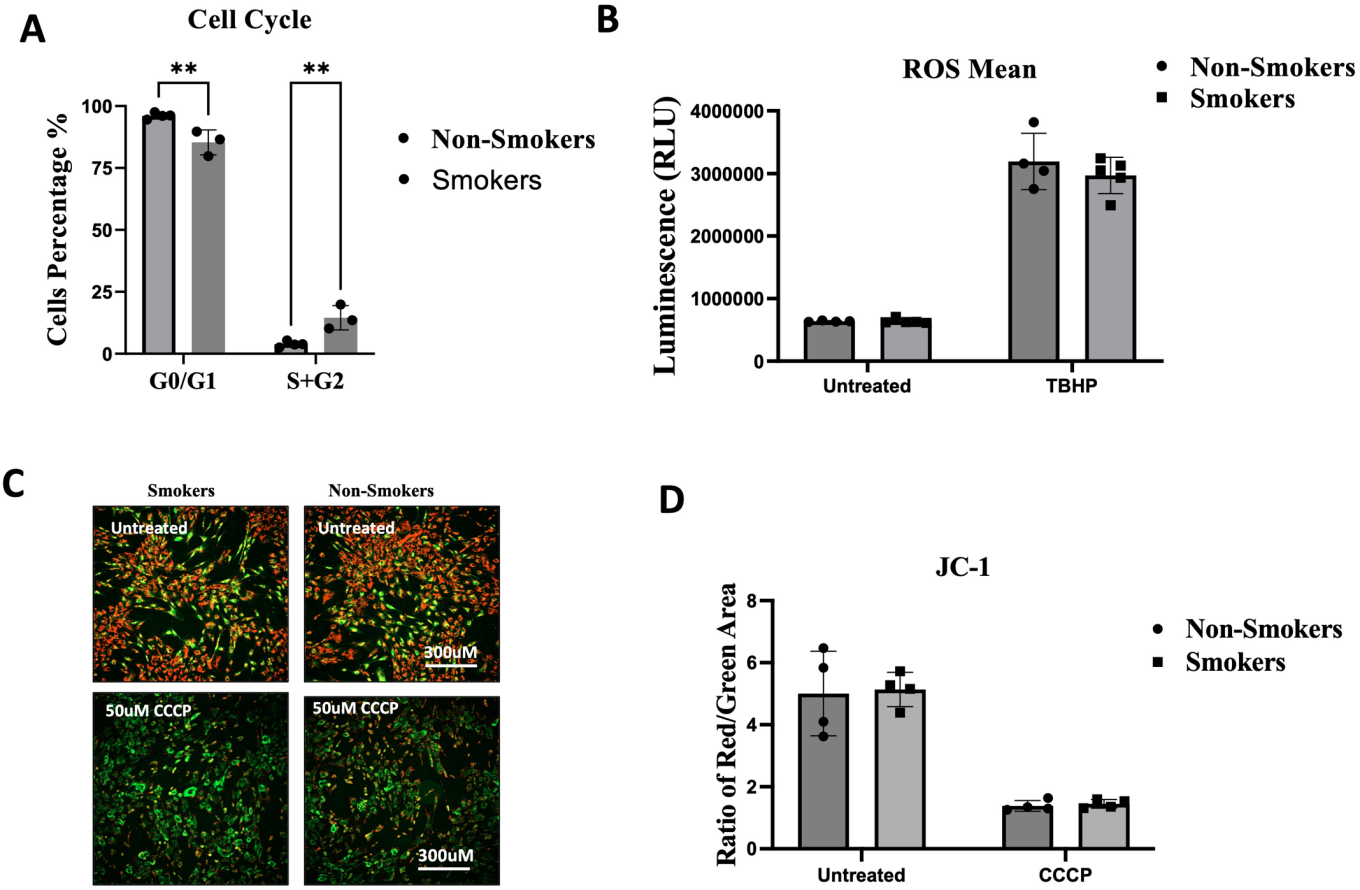
**The effect of smoking on cell cycle assessment of ADMSCs, ROS and MMP.** (A) Cell cycle analysis showed that the G0/G1 level of the cell cycle was significantly higher among the non-smoking group (**P<0.0010), while the S+G2 was significantly higher in the smoking group (**P<0.0010). (B) ROS median fluorescent intensity demonstrated no significant difference between smokers and non-smokers. (C) Fluorescent images represent the red aggregates (indicating healthy mitochondria) and the green monomers (indicating damaged mitochondria) in untreated samples compared to control CCCP-treated samples. (D) The red/green fluorescence ratio in untreated and CCCP-treated samples. scale bar: 500 µm, *P≤0.05.

Cell cycle analysis was conducted using Propidium Iodine (PI) stain, which binds to nucleic acids to measure the DNA content in the cells. G0/G1 ratio was significantly higher in the non-smoking group (*P*≤0.001), while the S+G2 was significantly higher in the smoking group (*P*≤0.001). Thus, the smokers’ ADMSCs spent more time in the S+G2 phase of the cell cycle (Fig. 2A). ROS levels were assessed using a luminescence-based assay with fluorescence detection performed at 488 nm excitation and 520 nm emission. Results revealed no significant difference in ROS production between ADMSCs isolated from smokers and non-smokers, implying comparable levels of oxidative stress in ADMSCs of both groups (Fig. 2B).

MMP was detected using a JC-1 fluorescent probe. JC-1 fails to aggregate in the matrix of mitochondria in impaired mitochondria due to the reduction or loss of MMP. Changes in MMP can be calculated by measuring the proportion of green fluorescence. The higher the proportion of green fluorescence, the lower the MMP level. Fluorescent images of ADMSCs showed no observed morphological difference between smokers and non-smokers (Fig. 2C). In our study, the red/green fluorescence ratio was higher in non-smokers’ ADMSCs than in smokers, with no significant difference, indicative of healthy mitochondria in both groups (Fig. 2D).

### Multilineage differentiation potential of ADMSCs

#### Osteogenic differentiation

**Fig. 3.**
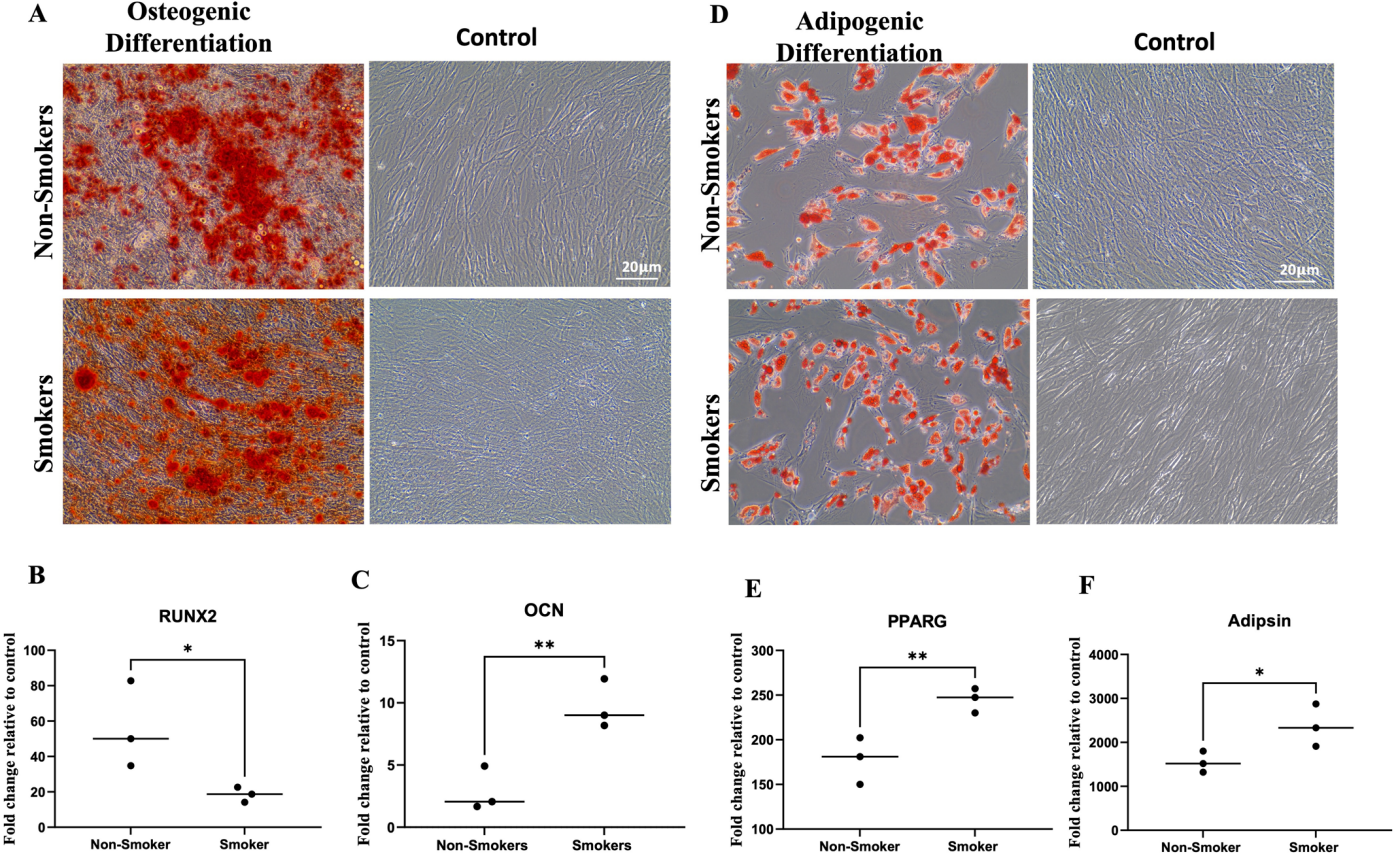
**Alizarin Red and Oil Red O staining of ADMSCs osteogenic differentiation cultures**. (A) Alizarin Red staining of the calcium deposition in smoking and non-smoking ADMSCs after 21 days of osteogenic differentiation, respectively. Control images represent cells maintained in the CCM without osteogenic induction. Magnification power 20Å∼; Scale bar: 20 μm. (B,C) qPCR-results of OCN and RUNX2 genes expression in smoking and non-smoking BM-MSCs. Scale bar: 20 μm. (D) Oil Red O staining of the oil granules in smoking and non-smoking ADMSCs after 28 days of adipogenic differentiation. (E,F) qPCR-results of PPARG and Adipsin genes expression in smoking and non-smoking ADMSCs.

Following successful expansion and morphological characterization, we assessed ADMSCs’ potential to differentiate into osteogenic and adipogenic lineages. Alizarin Red staining confirmed the successful differentiation of MSCs from both groups into osteogenic lineage after 21 days of induction. No noticeable morphological differences were detected between the two groups in the intensity of osteogenic cellular staining. Cells maintained in cell culture media showed the absence of Alizarin Red staining (Fig. 3A). qRT-PCR results confirmed the ability of these cells to differentiate towards the osteogenic lineage as the expression of osteogenic differentiation marker genes RUNX2 and OCN were increased following MSCs differentiation. OCN was significantly more expressed in ADMSCs from smokers, RUNX2 was more highly expressed in MSCs from non-smokers. This suggests that smoking may have divergent effects on different pathways within the osteogenic differentiation process, potentially enhancing certain aspects while inhibiting others (Fig. 3B,C).

#### Adipogenic differentiation

ADMSCs from both groups also successfully differentiated into the adipogenic lineage, as confirmed by the positive staining with Oil Red O staining of lipid vacuoles after 28 days of the induction process, with no apparent differences between the two groups. Cells maintained in cell culture media as controls showed the absence of Oil Red O staining (Fig. 3D). qRT-PCR results confirmed the ability of these cells to differentiate towards the adipogenic lineage as the expression of adipogenic differentiation marker genes PPARG and Adipsin were increased following adipogenic differentiation. Both genes were significantly more expressed in cells from the smoking group than from the non-smoking group. This indicates that cigarette smoking may promote a shift in MSC fate towards adipogenesis. Overall, our findings demonstrate that smoking differentially affects MSC differentiation potential, enhancing adipogenesis while showing a more complex, gene-dependent effect on osteogenesis (Fig. 3E,F).

### Oxidative stress gene expression analysis

Following RNA extraction from adipose tissue-derived MSC samples obtained from smokers and non-smokers, gene expression profiling was conducted using PCR arrays, with a standard 1.5-fold gene expression change set as the significance threshold. Out of 84 genes examined, twenty-five (30%) exhibited up- or downregulation by more than 1.5-fold. Among these, 13 genes were upregulated (Table S2), while 12 genes were downregulated, as shown in (Table S3).

The study of the gene expression revealed the upregulation of several key genes associated with oxidative stress and cellular response mechanisms, including *CYGB*, *DHCR24*, *GPX2*, *SOD3*, *TXNRD2*, *UCP2*, *FOXM1*, *HSP90AA1*, and *NOS2*. These genes indicate an increased cellular effort to mitigate oxidative stress, protect against oxidative damage, regulate mitochondrial function, promote cell proliferation despite stressful conditions, and manage protein integrity and inflammatory stress. Conversely, several genes were significantly downregulated, reflecting impaired cellular functions (Smirnov et al., 2016; Su et al., 2017; Ingold and Conrad, 2018; Shahid et al., 2020; Tsai et al., 2022), including *ALOX12*, *CCL5*, *DUOX1*, *DUSP1*, *GSTZ1*, *HMOX1*, *MB*, *SFTPD*, *BNIP3*, and *PTGS1***/***2*. These changes suggest reduced inflammatory signaling, impaired detoxification, altered cellular metabolism and immune function, and stress-induced alterations (Poss and Tonegawa, 1997; Zhang et al., 2007; Ameziane-El-Hassani et al., 2015; Shahid et al., 2020).

**Fig. 4.**
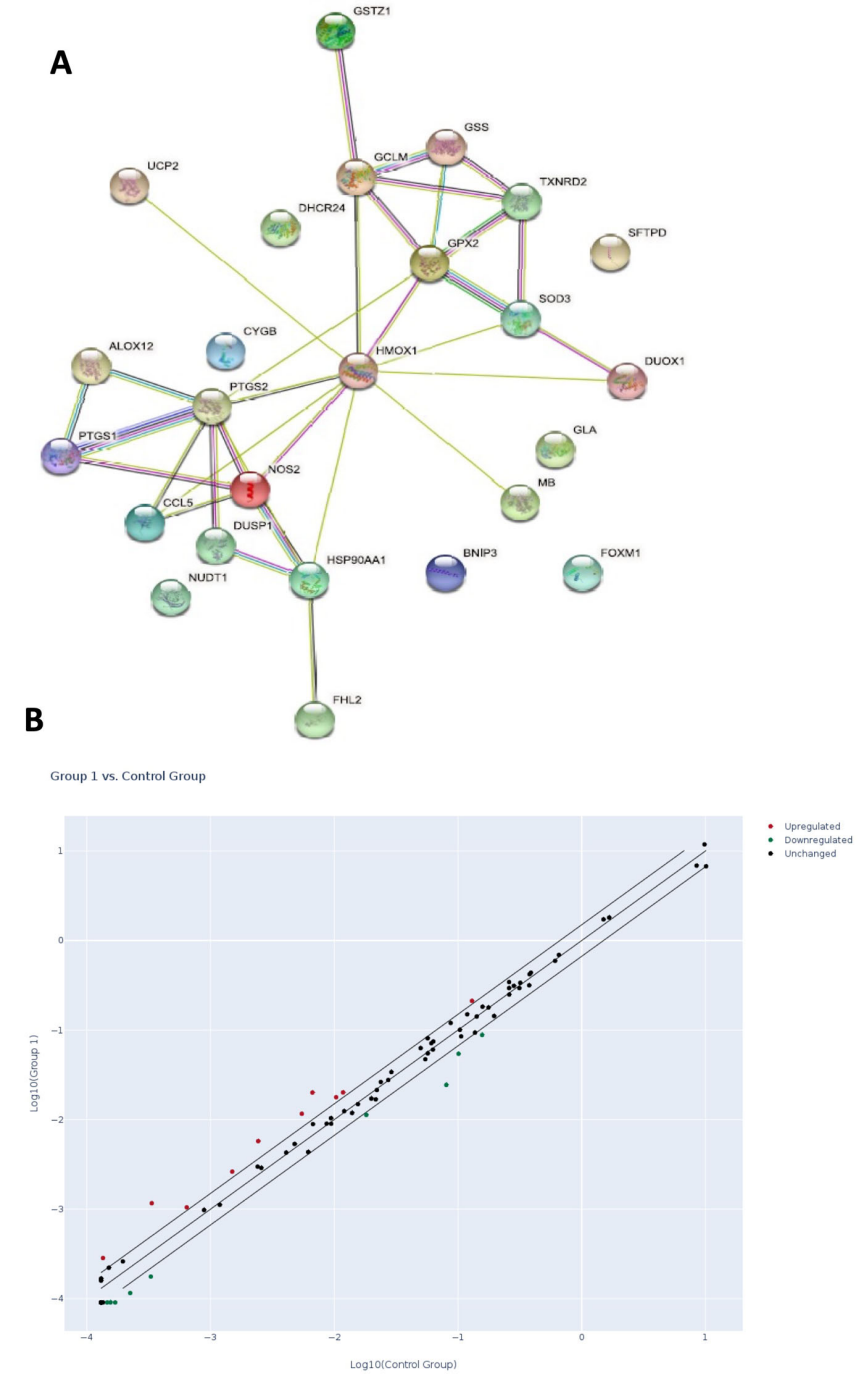
**Summary of gene expression analysis in smokers and non-smokers.** (A) The network connection between the upregulated and downregulated genes. (B) Scatter plot compares the normalized expression of every gene on the human oxidative stress PCR array between the two selected groups, the control, non-smokers group and the smokers group, by plotting them against each other to visualize gene expression changes. The center diagonal line indicates unchanged gene expression, while the outer lines indicate the selected fold regulation threshold. Genes with data points beyond the outer lines in the upper left and lower right corners are up- or downregulated, respectively, by more than the fold regulation threshold in the y-axis group relative to the x- axis group.

The network connection between the upregulated and downregulated genes and the plotted normalized expression of every gene on the human oxidative stress PCR Array between the two groups are shown in (Fig. 4)

## DISCUSSION

Mesenchymal stem cells (MSCs) are increasingly recognized as a viable treatment option in various clinical settings, with numerous clinical trials exploring their therapeutic potential across various conditions (Lou et al., 2021; Huang et al., 2022). Among the different sources of MSCs, the use of ADMSCs is rising due to their abundance and ease of collection (Naji et al., 2019). As the use of MSCs, particularly ADMSCs, continues to increase, it is essential to adhere to GMP standards to ensure the safety and effectiveness of these therapies. GMP guidelines address critical factors such as preventing contamination and maintaining genetic integrity, which are vital for the successful clinical application of MSCs (Sensebé et al., 2013). However, one important aspect that is often overlooked is the impact of smoking on MSCs. Exposure to cigarette smoke and nicotine can adversely affect the regenerative capacity and therapeutic efficacy of MSCs (Greenberg et al., 2017), highlighting the need to consider this factor in both preclinical and clinical studies.

In this study, ADMSCs were obtained from the adipose tissue of healthy individuals, both smokers and non-smokers, with no previous history of systemic diseases. These cells were cultured in 5% platelet lysate and displayed the characteristic fibroblast-like morphology. Additionally, ADMSCs did not express hematopoietic markers (CD34, CD11b, CD19, CD45, and HLA-DR) (ratio <0.5%) but were positive for CD90, CD105, CD44, and CD73 surface markers (ratio >90%), aligning with findings from previous studies (Dominici et al., 2006; Ababneh et al., 2022).

Previous studies have reported a decrease in MSC proliferation following exposure to smoking both *in vitro* and *in vivo*. A study by Wahl et al. demonstrated a significant reduction in the proliferation of ADMSCs following *in vitro* exposure to cigarette smoke extract​ (Wahl et al., 2016)​. Additionally, *in vivo* studies on smokers have shown comparable results. For instance, a study by Barwinska et al. found that ADMSCs isolated from smokers exhibited markedly reduced proliferation rates compared to non-smokers (Barwinska et al., 2018). Our study used the MTT assay to measure MSC proliferation under smoking conditions *in vivo* and showed a consistent decrease in proliferation, corroborating these previous findings (Barwinska et al., 2018).

Apoptosis assays in previous studies have shown varying effects of nicotine and smoking on MSCs. In a study by Zeng et al., nicotine treatment increased the number of apoptotic cells (Zeng et al., 2014). Conversely, another study found that nicotine treatment did not lead to significant deviations in cell viability as measured by apoptosis assay, suggesting that nicotine alone might not induce apoptosis in MSCs (Yang et al., 2017). In the first study, it was also found that nicotine treatment resulted in a decrease in MMP (Zeng et al., 2014). Our research found that the proportion of viable MSCs decreased in smokers, as indicated by the apoptosis assay. However, there was no significant change in MMP between smokers and non-smokers. This discrepancy between the viability and MMP results in our study suggests that factors other than mitochondrial dysfunction might contribute to smokers’ reduced cell viability.

The cell cycle analysis revealed a significant increase in the G0/G1 ratio in the non-smoking group and an accumulation of cells in the S and G2/M phases in the smoking group. While the S and G2/M phases are typically associated with active cell proliferation, the accumulation of cells in these phases in the smoking group likely reflects cell cycle delay or arrest. This is potentially due to stress and DNA damage caused by smoking, which activates DNA damage checkpoints and hinders efficient progression through mitosis. This finding aligns with the observed decrease in cell proliferation and viability. The increased stress response and the need for DNA repair in smoking MSCs, as evidenced by the upregulation of stress-related genes, likely contribute to the cell cycle arrest in the S+G2 phases.

The apoptosis assay results, showing a significantly lower proportion of viable cells in the smoking group, complement these findings. Nicotine exposure, as demonstrated by Zeng et al., induces apoptosis, supporting the increased cell death observed in the smoking group (Zeng et al., 2014). The absence of significant differences in ROS levels and mitochondrial membrane potential between controls and smokers suggests that while oxidative stress pathways are activated, the cells manage ROS levels to some extent. However, prolonged stress still leads to increased cell death.

In our study, while ADMSCs from smokers and non-smokers showed no morphological differences during adipogenic and osteogenic differentiation, significant variations in gene expression were observed. Adipsin and PPARG were more highly expressed in smokers, indicating enhanced adipogenic potential, consistent with Ng et al., who reported increased lipid production in MSCs derived from the periodontal ligament (PDLSCs) of smokers (Ng et al., 2015). However, Wahl et al. observed no significant effect of 0.5% CSE on adipogenic differentiation in ADMSCs (Wahl et al., 2016).

For osteogenesis, OCN was more expressed in smokers, while RUNX2 was higher in non-smokers. This aligns with findings of reduced calcium deposition and osteogenic marker expression in smoker periodontal ligament MSCs (Ng et al., 2015). Similarly, nicotine exposure has been shown to significantly reduce RUNX2, OCN, and other osteogenic markers in human bone marrow-derived MSCs (BMMSCs) and PDLSCs (Ng et al., 2013). On the other hand, Wahl et al. observed that 0.5% CSE did not significantly affect calcium deposition in BMMSCs after 20 days, though it did upregulate RUNX2 and OCN gene expression (Wahl et al., 2016).

Taken together, these findings highlight the complexity of the effects of smoking on MSC differentiation, where the mode of exposure [*in vivo* smoking versus *in vitro* cigarette smoke extract (CSE) or nicotine exposure] appears to play a crucial role in modulating the differentiation potential of MSCs.

Our results underscore the importance of considering smoking status in the clinical application of MSCs and the development of MSC-based therapies. While our study focuses on the impact of smoking, future research should consider a broader range of donor variables to comprehensively understand their influence on MSC quality. Further investigation is needed to elucidate the precise mechanisms by which smoking affects MSC function *in vivo* and to develop strategies that mitigate these effects, ensuring the safety and efficacy of MSC therapies in both smokers and non-smokers.

## MATERIALS AND METHODS

### Sample collection

This study obtained approval from the Institutional Review Board (IRB) committee (IRB Number: 2021/241). The donors signed written informed consent for their participation in the study. Adipose tissue aspirates were obtained from patients undergoing liposuction at the Plastic Surgery Department / Jordan University Hospital (JUH). This study enrolled five cigarette-smoking females and five non-smoking females with ages ranging between 35 and 50 years old, ensuring comparable age distribution between the two groups. All donors were undergoing liposuction primarily for cosmetic reasons rather than medical weight loss. Smokers were selected based on their history of consuming 15-30 cigarettes daily for approximately ten years the equivalent of 7.5-15 pack-years. A pack-year is a unit used to quantify the amount of smoking exposure over time, calculated as the number of packs of cigarettes smoked per day multiplied by the number of years the individual has smoked. All participants had no known systemic diseases or prolonged medication use, determined from their medical records. Samples were collected during liposuction surgery using sterile techniques by a plastic surgeon. Approximately 240 ml of adipose tissue were aspirated and divided into twelve 50 ml conical tubes.

### Isolation of MSCs from adipose tissue samples

The adipose tissue aspirates were divided into 50 ml conical tubes, 20 ml adipose tissue each. Then, 15 ml of 0.075% Collagenase type I (Worthington Biochemical, USA) were added to each tube to digest the extracellular matrix. The suspension was incubated for 30 min at 37°C in a CO_2_ incubator on a shaker. After that, 15 ml of 1× PBS (Gibco, USA) were added to each tube to neutralize the collagenase. The digested fat was then centrifuged at 1200×***g*** for 10 min. The resulting pellets were collected in two 50 ml conical tubes and filtered through a 70 µm cell strainer. Then, the resulting cell suspension was centrifuged at 500×***g*** for 10 min. The cell pellet was suspended in a 50 ml growth media of alpha minimal essential media (α-MEM) supplemented with 5% platelet lysate, 1% penicillin/streptomycin, and 2 mM L-Glutamine. Subsequently, the cells were counted and plated at a seeding density of 0.18×10^6^ cells/cm^2^. These cultures were maintained in complete culture media (CCM) at 37°C and 5% CO_2_. All samples were expanded for two to five passages and stored in liquid nitrogen for further use. The platelet lysate was derived from multiple platelet apheresis pools, previously processed in the blood banking unit at JUH. Following three temperature cycles, including freezing at −80°C and heating at 37°C, platelets were eliminated via centrifugation at 1400×***g*** for 10 min (Al-Kurdi et al., 2021).

### Flow cytometric immunophenotyping of MSCs

Cultured ADMSCs, isolated from smokers and non-smokers at passage 3, were trypsinized using 1× TrypLE (Invitrogen, USA), centrifuged, and resuspended at a concentration of 1×10⁶ cells in a staining buffer containing 2% bovine serum albumin (BSA) in PBS. A combination of fluorescently conjugated antibodies targeting MSC positive markers (CD90, CD105, CD73, and CD44) and MSC negative markers (CD34, CD45, CD14 CD11b, CD79a CD19, and HLA-DR), along with their respective isotype controls, were used according to the manufacturer's instructions. The results were analyzed using the BD Stem Flow hMSC Analysis kit (BD Biosciences, USA) processed on BD FACSCanto II equipment and then analyzed using BD FACSDiva software.

### Cell viability assays

#### MTT assay

To assess cellular viability, metabolic activity was measured using the MTT (3-(4,5-Dimethyl-2-thiazolyl)-2,5-diphenyl-2H-tetrazolium bromide, ATCC, USA) colorimetric assay. In passage 3, ADMSCs from both experimental groups were seeded into 96-well plates with a density of 10,000 cells per well. Following a 24-h incubation period, 10 µl of the MTT reagent was added to each well, and cells were incubated for four hours at 37°C. Subsequently, the MTT solution was aspirated, and 50 µl of dimethyl sulfoxide (DMSO) was added to dissolve the Formazan crystals. The absorbance was then measured at 570 nm using a plate-reader GloMax-Multi Detection System (Promega, USA) within a wavelength detection range of 450 to 650 nm. This process was repeated after 48 h.

### CFU assay

ADMSCs from smokers and non-smokers were seeded in duplicate sets at varying densities of 100, 200, and 300 cells/well in six-well plates and cultured for 10 days under standard culture conditions, with medium exchange every 3 days. Following incubation, cells were rinsed with 1× PBS, fixed with 100% methanol for 15 min, stained with 5% crystal violet for 5 min, washed twice with distilled water, and air-dried. The colonies were scored at a 4× lens of an inverted microscope (Primo Vert, Carl Zeiss, Germany). The following equation was used to determine the percentage of cells capable of forming colonies: number of colonies per well/ seeding density of the same well* 100%.

### Reactive oxygen species (ROS) level measurement

According to the manufacturer's instructions, the reactive oxygen species level was detected using the Total ROS Assay Kit 520 nm (Invitrogen, USA). For each sample, 7×10³ cells/well of passage 3 ADMSCs, derived from smokers or non-smokers, were seeded in white surface, tissue-culture-treated 96-well plates (Costar, Corning, USA) and incubated for 48 h at 37°C and 5% CO_2_ and then 1× ROS stain was prepared by adding 10 µl of 500× stock reagent to 5 ml prewarmed serum-free media. The CCM was aspirated, 50 µl of the 1× ROS stain solution was added, and the plates were incubated at 37°C for 60 min. For control wells, 200 µM tert-butyl hydrogen peroxide (TBHP) was prepared in serum-free media and added after the first 30 min. Following that, fluorescence detection was done at 488 nm excitation and 520 nm emission on Biotek Cytation 5, and the analysis was carried out using Bioteck Gen 5 data analysis software (BioTek, USA).

### Apoptosis detection by flow cytometry

Apoptosis was assessed using the Annexin V-FITC Apoptosis Detection Kit (Abcam, UK) following the manufacturer's protocol. PI was employed as a nucleic acid stain to identify dead cells in flow cytometry. At passage 3, cells were counted, and 5×10^5^ cells were re-suspended in 500 µl of the 1× binding buffer provided by the kit. Annexin V-FITC (5 µl) was added to the cells and incubated in darkness for 5 min. Subsequently, 5 µl of the PI staining solution (50 μg/ml) was added and incubated for another 5 min in darkness. Annexin V-FITC binding was analyzed using the BD FACSCanto II instrument (BD, USA) with excitation at 488 nm and detection at 530 nm using the FITC signal detector (FL1). PI staining was detected by the phycoerythrin emission signal detector (FL2). Sample analysis was done using BD FACSDiva software (BD, USA).

### Cell cycle analysis

Cell cycle analysis was conducted using flow cytometry on ADMSCs from both study groups at passage 3. Cells were detached using trypsin, washed with PBS, and fixed in ice-cold 70% ethanol, stored at −20°C overnight. Upon thawing, cells were washed, centrifuged, and rehydrated in PBS for 15 min. Subsequently, the cell pellet was suspended in a solution containing 50 µg/ml PI stain and incubated for 15 min at room temperature. PI binding analysis was performed using the BD FACSCanto II instrument (BD, USA) with excitation at 535 nm and detection at 617 nm. Sample analysis was done using BD FACSDiva software (BD, USA).

### Mitochondrial membrane potential (MMP)

The MMP was assessed using the MitoProb™ JC-1 Assay Kit (Invitrogen, USA). For this procedure, 7×10^3^ cells per well were plated in black, tissue culture-treated 96-well plates (Costar, Corning, USA) and incubated for 48 h. Subsequently, each well was treated with 100 µl of a 2 µM solution of JC-1 (5′,6,6′-tetrachloro-1,1′,3,3′-tetraethylbenzimidazolylcarbocyanine iodide) prepared in serum-free medium and incubated for 60 min at 37°C in a 5% CO2 atmosphere. During the last 30 min of incubation, 50 µM CCCP (carbonyl cyanide 3-chlorophenylhydrazone) was added to control wells, then cells were rinsed with PBS. Fluorescence intensities were then recorded using Biotek Cytation 5 (BioTek, USA) at an excitation wavelength of 485 nm and emission wavelength of 528 nm for green J-Monomers and excitation at 535 nm with emission at 590 nm for Red J-aggregates. Images were captured using BioTek Gen 5 data analysis software (BioTek, USA). The ratio of red to green fluorescence was calculated by dividing the fluorescence intensity of the red regions marking healthy cells by that of the green regions marking unhealthy or apoptotic cells, with the analysis performed using BioTek Gen5 data analysis software (BioTek, USA).

### Differentiation of MSCs

#### Osteogenic differentiation

Cells in passage 3 from both groups (smoking and non-smoking) were used in osteogenic differentiation. Briefly, cells were plated onto six-well plates at a seeding density of 4×10^3^ cells/cm^2^. Upon reaching 50% confluence, CCM was switched to the osteogenic differentiation medium (Invitrogen, USA), while fresh CCM media was used to control undifferentiated wells. Cells were stained with Alizarin Red staining on day 21 and subsequently viewed and imaged using the EVOS XL Core Imaging System supplied with 3.1 MP color camera with integrated software (Thermo Fisher Scientific, USA).

#### Adipogenic differentiation

Cells from both groups were also used in osteogenic differentiation. Cells were plated onto six-well plates at a seeding density of 4×10^3^ cells/cm^2^. Upon reaching 50% confluence, CCM was switched to the complete adipogenic differentiation medium, while fresh CCM media was used for the control wells. On day 28, cells were stained with Oil Red O staining and subsequently viewed and imaged using the EVOS XL Core Imaging System supplied with 3.1 MP color camera with integrated software (Thermo Fisher Scientific, USA).

### Quantitative RT-PCR

RNA extraction was performed from cell pellets of either osteogenic or adipogenic differentiation using RNeasy® Mini Kit (Qiagen, Germany) following the manufacturer's instructions. RNA concentration was measured on a NanoDrop 8000 Spectropotometer (ThermoFisher, USA). This was followed by cDNA synthesis using 1 µg RNA in a 20 µl total volume using PrimeScript™ RT Master Mix (Takara, Japan).

Quantitative RT-PCR was performed using TB Green^®^ Premix EX Taq™ II (Tli RNase H Plus) kit (Takara, Japan) in Bio-Rad CFX96 cycler (Bio-Rad, USA). Specific primers were used to amplify OCN 2, RUNX, LPL and PPAR-γ. GAPDH was used as a housekeeping gene, and cDNA samples were diluted to 10 ng/µl. Samples were run in triplicates in a 96-well qRT-PCR plate. The cycling conditions were as follows: first step of initial denaturation: 95°C for 30 s, then 40× of the seconf step: 95°C for 5 s and the third step for 1 min. All primer sequences and their optimal annealing temperatures are described in Table S1.

### Human oxidative stress gene expression assay

To evaluate the effect of cigarette smoking on the oxidative stress signaling pathway, a PCR array was performed on the ADMSCs from smokers and non-smokers. Total RNA was extracted from the ADMSCs of both groups using the RNeasy Mini Kit according to the manufacturer's instructions (Qiagen, USA). The RNA concentration was measured on a NanoDrop 8000 Spectrophotometer (ThermoFisher Scientific, USA). A 0.5 µg extracted RNA was transformed to cDNA using the RT2 First Strand Kit (Qiagen, USA). Then, cDNA samples were diluted and amplified with the RT2 SYBR^®^ green master mix of (PAHS-065Y, RT2 Profiler™ Human Oxidative Stress Plus PCR Array, Qiagen, USA) according to the manufacturer's instructions, and loaded into the 96-well plate of the array. The amplification conditions were performed as follows: 95°C for 10 min, then 40 cycles of 95°C for 15 s and 60°C for 1 min. Samples were run on the CFX 96 C1000 system (Biorad, USA). Data were analyzed automatically, according to the SABiosciences company (Qiagen, USA) web portal, www.SABiosciences.com/pcrarraydataanalysis.php, by using the ΔCt method. The expression levels of the genes were normalized to the following housekeeping genes: beta-2-microglobulin (B2M), hypoxanthine phosphoribosyl transferase 1 (HPRT1), ribosomal protein lateral stalk subunit P0 (RPLP0) and glyceraldehyde-3-phosphate dehydrogenase (GAPDH). The differential expression level of the oxidative stress genes was identified for data analysis. A cut-off point of 1.5 was used as a threshold to determine the statistical significance of up- or downregulated genes.

The PCR mix was prepared by combining 1350 µl of 2× RT2 SYBRR Green Mastermix, 102 µl of cDNA synthesis reaction mix, and 1248 µl of RNase-free water, resulting in a total volume of 2700 µl. Subsequently, 25 µl of the PCR mix was dispensed into each RT2 Profiler PCR Array plate well using a 12-channel XLS pipette (Mettler-Toledo Rainin, USA). The plate was securely sealed with optical thin-wall eight-cap strips to prevent contamination. After sealing, the RT^2^ Profiler PCR Array plate underwent centrifugation for 1 min at 1000×***g*** at room temperature to eliminate bubbles. Each well's cycle was automatically calculated using the real-time cycler software. Subsequent data analysis was done using the online software of QIAGEN's GeneGlobe Data Analysis Center.

### Statistical analysis

All experiments detailed in this study were conducted independently on a minimum of three occasions. Data was analysed using GraphPad Prism 8 (GraphPad Software, San Diego, CA, USA) and presented as mean±standard deviation. Statistical comparisons between groups were performed utilising multiple *t*-tests. Significance levels were set at *P*-values ≤0.0500 (*), ≤0.0010 (**), ≤0.0001 (***), and ≤0.00001 (****). Experiments were performed at least three times, each with triplicate samples. The sample size (*n*=3) refers to both biological replicates and technical replicates.

## Supplementary Material

10.1242/biolopen.061665_sup1 Supplementary information
