# Supplementary material for: Effect of cigarette smoke on the proliferation, viability, gene expression, and cellular functions of adipose-derived mesenchymal stem cells from smoking and non-smoking donors
Source: Biol Open. 2024 Dec 3;13(12):bio061665. doi: 10.1242/bio.061665 (PMC11646114; doi:10.1242/bio.061665)
Supplement: Supplementary information [file biolopen-13-061665-s1.pdf]

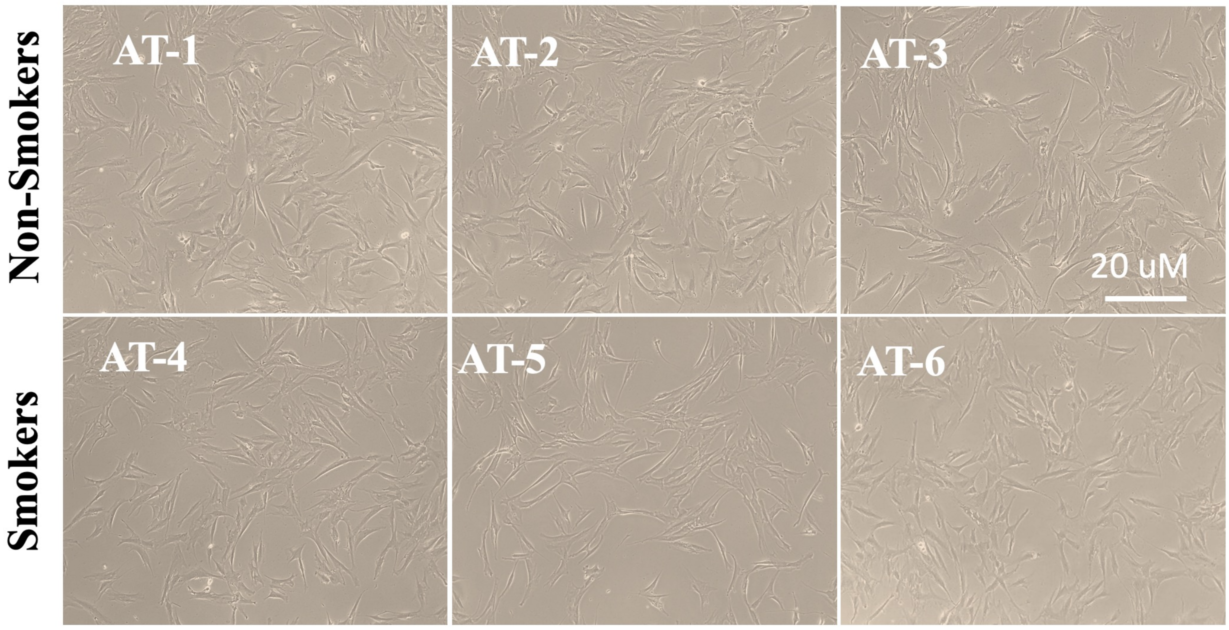

Fig. S1. Morphological observation of smokers and non-smokers-derived ADMSCs.

Table S1. Prime sequences and their optimal annealing temperatures

Gene	Primer sequences	Annealing temperature
OCN 2	F: 5′ AGCTCAATCCGGACTGT 3′,<br>R: 5′GGAAGAGGAAAGAAGGGTGC	60°C
RUNX2	F: 5′ ATGTGTGTTTGTTTCAGCAGCA 3′,<br>R: 5′ TCCCTAAAGTCACTCGGTATGTGTA 3′	60°C
PPAR -γ	F: 5′ TGAACGACCAAGTAACTCTCC 3′,<br>R: 5′ CGCAGGCTCTTTAGAAACTCC 3′	60°C
Adipsin	F: CCCGCCCGTGGTGTTATTTA<br>R: CACGCGTTCAGCCATGTTTA	60°C
GAPDH	F: 5’CCTGTTCGACAGTCAGCCG-3’<br>R: 5’CGACCAAATCCGTTGACTCC-3’	58°C–60°C

Table S2. List of the upregulated genes associated with oxidative stress and their physiological function. Gene expression in smoking ADMSCs compared to non-smoking ADMSCs Control group.

Gene symbol	Gene Description	Gene function	Fold of Regulation
CYGB	Cytoglobin	Have protective function during conditions of oxidative stress, involved in oxygen transport	(+) 1.75
DHCR24	24-dehydrocholesterol reductase	Catalyzes the reduction of the delta-24 double bond of sterol intermediates and protects cells from oxidative stress	(+) 1.72
FOXM1	Forkhead box M1	The transcriptional factor that regulates the expression of cell cycle genes is essential for DNA replication and mitosis and plays a role in controlling cell proliferation.	(+) 6.26
GPX2	Glutathione peroxidase 2	Protect cells against oxidative damage, catalyze the reduction of organic hydroperoxides and hydrogen peroxide by glutathione	(+) 2.11
NUDT1	Nudix hydrolase 1	Acts as a sanitizing enzyme for oxidized nucleotide pools, thus suppressing cell dysfunction and death induced by oxidative stress	(+) 2.37
SOD3	Superoxide dismutase 3	Antioxidant enzyme that catalyzes the conversion of superoxide radicals into hydrogen peroxide and oxygen, which may protect the tissues from oxidative stress	(+) 1.71
TXNRD2	Thioredoxin reductase 2	Implicated in the defenses against oxidative stress, it encodes a mitochondrial form important for scavenging reactive oxygen species in mitochondria	(+) 1.63
UCP2	Uncoupling protein 2	Separate oxidative phosphorylation from ATP synthesis with energy dissipated as heat	(+) 3.48
FHL2	Four and a half LIM domains 2	A molecular transmitter linking various signaling pathways to transcriptional regulation	(+) 1.63
GLA	Galactosidase alpha	Detoxification of a wide variety of reactive electrophiles	(+) 2.13
GSS	Glutathione synthetase	Belongs to the eukaryotic GSH synthase family. Glutathione is essential for a variety of biological functions, including the protection of cells from oxidative damage by free radicals, detoxification of xenobiotics, and membrane transport	(+) 1.51
NOS2	Nitric oxide synthase 2	Produces nitric oxide (NO), a reactive free radical which is a messenger molecule with diverse functions throughout the body	(+) 2.45
HSP90AA1	Heat shock protein 90 alpha family class A member 1	Promotes the maturation, structural maintenance and proper regulation of specific target proteins involved, for instance, in cell cycle control and signal transduction	(+) 1.61

A 1.5-fold change in expression was used as an arbitrary cut-off value; ADMSCs: Adipose tissue-derived mesenchymal stem cells.

Biology Open • Supplementary information

Table S3. List of the downregulated human oxidative stress-associated genes and their function. Gene expression in smoking ADMSC compared to non-smoking ADMSC group.

Gene symbol	Gene Description	Gene function	Fold of Regulation
ALOX12	Arachidonate 12-lipoxygenase	Catalyzes the stereo-specific peroxidation of free and esterified polyunsaturated fatty acids, generating a spectrum of bioactive lipid mediators	(-) 1.61
CCL5	C-C motif chemokine ligand 5	Involved in immunoregulatory and inflammatory processes	(-) 1.71
DUOX1	Dual oxidase 1	Generates hydrogen peroxide, which is required for the activity of thyroid peroxidase	(-) 1.94
DUSP1	Dual specificity phosphatase 1	play an important role in the human cellular response to environmental stress as well as in the negative regulation of cellular proliferation	(-) 4.03
GSTZ1	Glutathione S-transferase zeta 1	Detoxification of electrophilic molecules, including carcinogens, mutagens, and several therapeutic drugs, by conjugation with glutathione	(-) 1.61
MB	Myoglobin	Primarily responsible for the storage and facilitated transfer of oxygen from the cell membrane to the mitochondria, it also plays a role in regulating physiological levels of nitric oxide	(-) 1.86
SFTPD	Surfactant protein D	This gene is part of the innate immune response, protecting the lungs against inhaled microorganisms and chemicals	(-) 1.87
GCLM	Glutamate-cysteine ligase modifier subunit	is the first rate-limiting enzyme of glutathione synthesis	(-) 1.86
HMOX1	Heme oxygenase 1	Essential enzyme in heme catabolism	(-) 1.78
BNIP3	BCL2 interacting protein 3	Apoptosis-inducing protein that can overcome BCL2 suppression, Involved in mitochondrial quality control in response to mitochondrial damage	(-) 1.53
PTGS1	Prostaglandin-endoperoxide synthase 1	Regulates angiogenesis in endothelial cells	(-) 5.12
PTGS2	Prostaglandin-endoperoxide synthase 2	The key enzyme in prostaglandin biosynthesis, it acts both as a dioxygenase and as a peroxidase	(-) 2.20

A 1.5-fold change in expression was used as an arbitrary cut-off value; ADMSCs: Adipose tissue-derived mesenchymal stem cells.
